# Epiploic Appendagitis in a Young Adult Female: A Rare Cause of Acute Abdominal Pain

**DOI:** 10.7759/cureus.73953

**Published:** 2024-11-18

**Authors:** Paige O Daly, Kaylie C Ward, Joy Elliott

**Affiliations:** 1 Research, Edward Via College of Osteopathic Medicine, Blacksburg, USA; 2 Family Medicine, Edward Via College of Osteopathic Medicine, Blacksburg, USA

**Keywords:** acute abdominal pain, conservative management, ct imaging, epiploic appendagitis, gastrointestinal disorders, inflammatory conditions, misdiagnosis, young female

## Abstract

Epiploic appendagitis (EA) is an uncommon and frequently misdiagnosed cause of acute abdominal pain, typically affecting middle-aged males. This case report presents an atypical occurrence in a 32-year-old Hispanic female who presented with left lower quadrant pain, initially suspected to be diverticulitis or infectious colitis. Contrast-enhanced CT imaging revealed the characteristic findings of EA, a diagnosis that is rarely seen in young adult females. The patient was successfully treated through conservative measures and avoided unnecessary surgical interventions. This case emphasizes the importance of considering EA in the differential diagnosis of acute abdominal pain across diverse patient demographics. It also highlights the crucial role of contrast-enhanced CT imaging to accurately diagnose and treat EA. By increasing awareness of this condition among clinicians, we can improve diagnostic accuracy, reduce unnecessary interventions, and optimize patient care in cases of acute abdominal pain.

## Introduction

Epiploic appendagitis (EA) is a rare, benign, and self-limiting inflammatory condition affecting the epiploic appendages of the colon. These small, fat-filled sacs protrude from the serosal surface of the colon and can become inflamed due to torsion or thrombosis of the central draining vein [1]. Despite its benign nature, EA often presents as acute abdominal pain, mimicking more serious conditions such as acute diverticulitis or appendicitis, leading to frequent misdiagnosis and potential over-treatment.

The incidence of EA is relatively low, accounting for approximately 1% of all cases of acute abdominal pain in adults and 2% of cases initially diagnosed as acute appendicitis or diverticulitis [2-4]. Studies suggest that EA typically affects individuals between 40-50 years of age, with a higher prevalence seen in males and overweight individuals [2]. However, as this case report will demonstrate, EA can occur in atypical demographics, highlighting the need for increased awareness across all patient populations.

The nonspecific clinical presentation of EA poses a significant diagnostic challenge for clinicians. Patients often present with localized abdominal pain, most commonly in the left lower quadrant, although right-sided pain can also occur. Associated symptoms may include mild fever, nausea, and changes in bowel habits. The absence of specific laboratory markers further complicates the diagnostic process, emphasizing the crucial role of imaging studies, particularly CT scans, in accurately identifying this condition.

Misdiagnosis of EA can lead to unnecessary hospitalizations, antibiotic administration, and even surgical interventions, all of which carry inherent risks and increase healthcare costs. Therefore, a thorough understanding of EA, its presentation, and its diagnostic features is essential for all clinicians involved in the evaluation and management of acute abdominal pain.

This case report presents an atypical occurrence of EA in a young Hispanic adult female, stressing the importance of considering this diagnosis across diverse patient demographics. By examining the clinical presentation, diagnostic approach, and management of this case, we aim to increase awareness of EA among healthcare providers, potentially improving diagnostic accuracy and patient outcomes in similar cases of acute abdominal pain.

## Case presentation

A previously healthy 32-year-old Hispanic female presented to the emergency department with a three-day history of progressive left lower quadrant abdominal pain. The patient reported mild nausea, diarrhea, and decreased appetite along with her abdominal pain. She denied any vomiting, dysuria, hematuria, or vaginal bleeding/discharge.

Her medical history was unremarkable, with no chronic conditions or previous surgeries. She denied recent travel, changes in diet, or sick contacts. The patient had no known drug allergies, and her family history was non-contributory.

Vital signs were within normal limits, except for blood pressure: 159/85. On physical examination, the patient appeared uncomfortable but not in acute distress. Her abdomen was soft with significant tenderness, guarding, and rebound in the left lower quadrant. No palpable masses or organomegaly were noted. Laboratory findings revealed a slight leukocytosis.

The contrast-enhanced CT showed no evidence of pleural effusion or abdominal pericardial effusion. There was no lung mass, nodule, or pulmonary infiltrates. The liver, spleen, pancreas, adrenal glands, and kidneys were otherwise unremarkable. There was no retroperitoneal or mesenteric lymphadenopathy. There were no ascites or abdominal free air. The gallbladder was partially distended without abnormality. The small bowel appeared normal, and the appendix appeared normal. The colon was unremarkable, and the bladder was not distended. There was no pelvic mass or pelvic free fluid.

**Figure 1:**
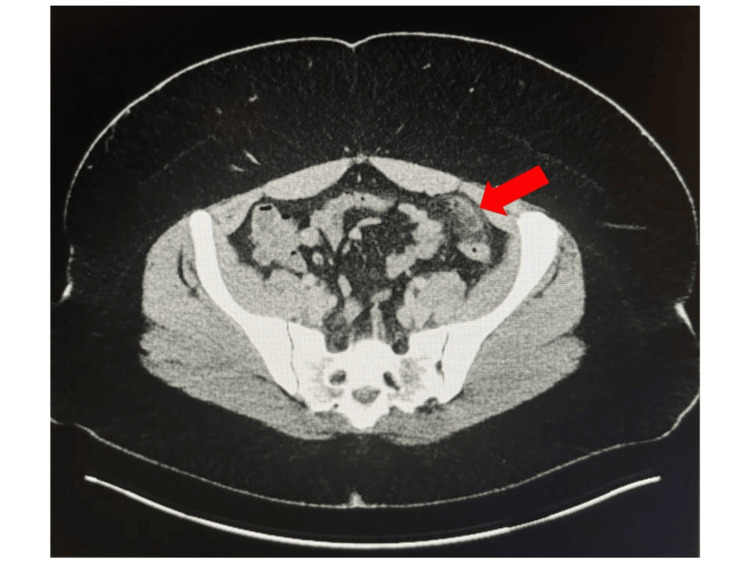
Axial contrast-enhanced CT image of the abdomen in a 32-year-old Hispanic female presenting with left lower quadrant pain The image demonstrates a characteristic oval-shaped fat-density lesion (red arrow) adjacent to the sigmoid colon. The lesion is surrounded by a hyperattenuating ring, consistent with the diagnosis of EA. Note the inflammatory changes in the surrounding fat, indicative of local inflammation. This imaging finding, often referred to as the "central dot sign," is pathognomonic for EA and crucial for differentiating it from other causes of acute abdominal pain.

**Figure 2:**
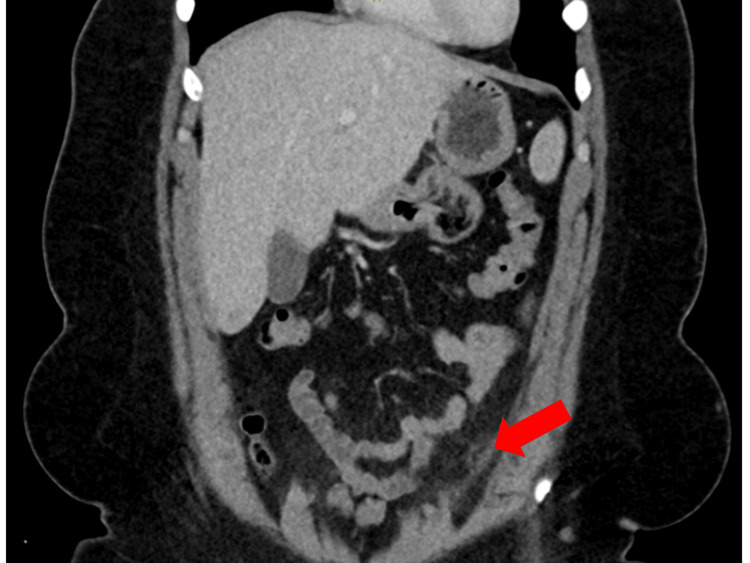
Coronal contrast-enhanced CT image of the abdomen in a 32-year-old Hispanic female presenting with left lower quadrant pain The image demonstrates a characteristic oval-shaped fat-density lesion (red arrow) adjacent to the sigmoid colon. The lesion is surrounded by a hyperattenuating ring, consistent with the diagnosis of EA.

Abnormal findings on the contrast-enhanced CT imaging showed a characteristic oval fatty mass with a hyperattenuated ring sign, adjacent to the proximal sigmoid colon in the anterior left lower quadrant, confirming the diagnosis of EA (Figures 1, 2). This imaging finding, known as the “central dot sign,” is crucial for differentiating EA from other causes of acute abdominal pain [4-6].

Based on the CT findings, a general surgeon was consulted to discuss treatment management. The surgeon confirmed that EA is typically a non-surgical condition, and conservative management, including anti-inflammatories and other pain control methods, could be initiated. The patient’s symptoms improved significantly over the next few days, and she was discharged with instructions for outpatient follow-up. During follow-up, a CT abdomen with and without contrast was recommended to monitor the condition and determine if the EA had completely resolved. The patient was educated about EA and advised to return if symptoms worsened or new symptoms developed; however, she did not follow up on the ordered CT.

## Discussion

Atypical demographic

This case of EA in a 32-year-old Hispanic female is noteworthy for its atypical demographic, as EA is more commonly seen in middle-aged males. A study by Son et al. reported a mean age of 52.6 years among EA patients, highlighting the rarity of this condition in younger adult females [7].

Diagnostic challenges

The patient's presentation closely mimicked more prevalent conditions such as diverticulitis or infectious colitis, which poses a significant diagnostic challenge. EA is frequently misdiagnosed as acute diverticulitis or acute appendicitis, underscoring the importance of maintaining a broad differential diagnosis when evaluating acute abdominal pain. Due to the differentials being more severe, this typically results in unnecessary hospitalizations, administration of antibiotics, and even unwarranted surgical interventions [4]. A study conducted by Rao et al. found that 11 out of 660 subjects were initially diagnosed with acute diverticulitis or acute appendagitis, and eight out of the 11 were misdiagnosed and underwent unnecessary hospitalizations or pharmaceutical therapy [3]. Another study concluded that 26.4% of EA cases are misdiagnosed and undergo unnecessary surgery [2]. That being said, it indicates that patients who have EA are at high risk for unnecessary surgeries or pharmaceutical therapy, which can lead to increased risk of short- and long-term complications.

Importance of imaging

The characteristic CT findings of an oval fatty mass with surrounding inflammation are crucial in establishing the correct diagnosis in this case. Correctly identifying the EA on the contrast-enhanced CT minimizes unnecessary treatments and is the core of quaternary prevention.

Effectiveness of conservative management

This case exemplifies the effectiveness of conservative management for EA, which aligns with current best practices. Surgical consultation confirmed that EA is a non-surgical condition, allowing for a treatment approach focused on anti-inflammatory medications and pain control. This approach is supported by multiple studies, including a retrospective analysis by Schnedl et al. involving 49 cases of EA, all of which were successfully managed conservatively [8]. Overall, this case highlights the need for increased awareness of EA among clinicians, which can enhance patient care and reduce healthcare costs associated with misdiagnosis and over-treatment.

Future research

Future research in EA should focus on several key areas to enhance our understanding and management of this condition. Developing clinical prediction models to aid in the early identification of EA is crucial, as it could reduce the reliance on advanced imaging and unnecessary management.

Additionally, studies investigating the potential role of patient demographics and risk factors in the development of EA could provide valuable insights into this rare condition. These studies might reveal previously unrecognized patterns or risk factors, particularly in atypical cases, similar to the female patient in this case report.

Finally, assessing the cost-effectiveness of various diagnostic and management strategies for suspected EA could inform policy decisions and clinical practice guidelines, leading to more efficient use of healthcare resources in the management of acute abdominal pain.

## Conclusions

EA, while relatively rare, should be an essential consideration in the differential diagnosis of acute abdominal pain, regardless of the patient's age or gender. This case report demonstrates that EA can occur in atypical demographics, such as young adult females, and emphasizes the need for heightened clinical awareness. Increased recognition of EA among healthcare providers can lead to more accurate diagnoses, reduced healthcare costs associated with misdiagnosis and over-treatment, and improved patient outcomes. The pivotal role of contrast-enhanced CT imaging in accurately diagnosing EA is evident, as it allows for differentiation from more common acute abdominal conditions.

As our understanding of EA continues to evolve, future research should focus on developing clinical prediction models for early identification, assessing long-term outcomes in conservatively managed patients, and investigating potential risk factors across diverse patient populations. By continuing to study and raise awareness of EA, we can enhance our ability to provide optimal, cost-effective care for all patients presenting with acute abdominal pain.
